# Analog of multiple electromagnetically induced transparency using double-layered metasurfaces

**DOI:** 10.1038/s41598-020-65418-x

**Published:** 2020-05-21

**Authors:** Siyuan Liu, Zhixia Xu, Xiaoxing Yin, Hongxin Zhao

**Affiliations:** State Key Laboratory of Millimeter Waves, Southeast University, Nanjing, 210096 China

**Keywords:** Materials for devices, Materials for energy and catalysis, Materials for optics, Structural materials

## Abstract

We reported an analog of electromagnetically induced transparency (A-EIT) featured by double transparent peaks in the spectrum. The A-EIT is realized by double-layered metasurface which consists of spoof localized surface plasmons (S-LSP) and cut-wire (CW)-square rings (SR) hybrid. Electric and magnetic S-LSP are excited as bright and dark modes respectively then couple with resonant modes of CW and SR simultaneously to achieve multiple A-EIT. Two bright modes of the electric S-LSP and SR are excited by external electric field directly that produce a bright-bright mode A-EIT. Moreover, the magnetic S-LSP, which cannot be excited by external field directly, is excited through near field coupling from CW, inducing another bright-dark mode A-EIT. Theoretical analysis with corresponding experiment in microwave band are introduced for better insights into physical essence of the double-peaks A-EIT.

## Introduction

EIT is a phenomenon of quantum destructive interference in three-level atomic systems and alters an absorptive medium transparency^1,2^. The transparency peaks are usually accompanied by high Q-value and intense dispersion, which has many potential applications, such as optical storage^3^, nonlinearity enhancement^4^, and ultrasensitive sensors^5^. EIT effects can occur not only in the systems that support quantum mechanics but also in the mechanical or others oscillator systems. Researchers had found that the A-EIT phenomena can arise in lots of classical coupled Lorentz oscillator systems, such as coupled attenuators^6^, waveguide resonators^7^, and optomechanical systems^8^. Metamaterials are heterogeneous hybrid materials that are handed to obtain extraordinary features resulting from hybrids of its structure and configuration, and the periodically-structure artificial unit cells are designed to be smaller than the wavelength of incident electromagnetic wave^9–12^. So the metamaterial is one of non quantum classical systems mimicking the EIT behavior. In past years, people had finished a series of extended works to metamaterial A-EIT, including all-dielectric 3D metamaterial, polarization tunable, superconducting A-EIT and so on^13–15^. Besides, Fano resonance is a kind of scattering resonance phenomenon that can produce asymmetric spectrum which having same characteristic of slow-light to the EIT phenomenon^16,17^ and attracting a lot of attentions by the researches. Coupling between the modes with almost the same resonant frequency is the necessary condition to obtain classical EIT in metamaterials. Therefore, the extent to phase of changes for A-EIT is negatively correlated with the difference of frequencies for two modes and positively correlated with the difference of Q-values. The bright mode, usually presented as low Q-value, is a resoance which can be directly excited by external fields. In contrast, the dark mode, presented as high Q-value mode in general, cannot be excited by external fields directly, just only be near-field coupled from the bright mode. Thus, two coupling ways can be utilized to generate an A-EIT: bright-dark mode coupling^17,18^ and bright-bright (quasi dark) mode coupling^19–21^. It is obvious that ‘dark-dark mode’ is unable to achieve the A-EIT because at least one bright mode must to be coupled energy by external incident electromagnetic waves. Besides, to all resonators, whether bright or dark mode, are utilized to establish a coupled Lorentz oscillator model, is the core for analysing an A-EIT in metamaterials. In these A-EIT metasurfaces, the plasmonic structures are widely applied due to the strong response on metal surface at the optical band^22,23^.

The spoof localized surface plasmons (S-LSP) can be classified by magnetic S-LSP^24^ and electric S-LSP^25^. There are two kinds of ways to distinguish them: one is comparing the S-parameters of S-LSP under the different external polarization, other one is observing their surface field distributions corresponding to each mode. However, it is hard to realize mimicking surface-plasmon on metal under the low frequency in the past, until the appearance of spoof surface plasmons (SSPs), which expands the study range of SP (surface plasmons) to terahertz^26^ and microwave band. We have noticed that magnetic S-LSP was recently proposed as relative to electric S-LSP, and magnetic S-LSP usually cannot be generated from the metasurface alone but in pairs with electric S-LSP^19,27,28^. On the other hand, a simple SSPs structure by prism-coupled bi-layered slit arrays was comprehensively proposed to realize an EIT analog^27–31^. The novel structure composes of prism-coupled bi-layered arrays with symmetric and antisymmetric resonances, inducing a transparency window.

Compared to the enlightened pioneering work realizing one transparent peak based on magnetic resonances^32^, we propose a novel A-EIT featured by double transparent peaks based on a bright-dark mode between CW and magnetic S-LSP and a bright-bright mode between electric S-LSP and SR. The excited electric S-LSP and magnetic S-LSP from spiral metasurface structure are simultaneously regarded as coupling modes for A-EIT realizing. Due to the proposed A-EIT need to the S-LSP exciting, it can also be considered as plasmon induced transparency (PIT). The multiple A-EIT has potential applications in the optical computing, polarization conversion and communications in highly integrated optical circuits^33–41^. In this works, the CW, SR and electric S-LSP, acting as bright modes, can be excited by the external field, while only the magnetic S-LSP is a dark mode which is unable to be excited by external field directly. We analyze the proposed metasurface using coupled Lorentz oscillator models, then simulations and experiments match well. Moreover, we numerically analyze the potential sensing applications of the double-peaks mode A-EIT for permittivity of the surrounding environment.

## Bi-layered metamaterials structure design

**Figure 1:**
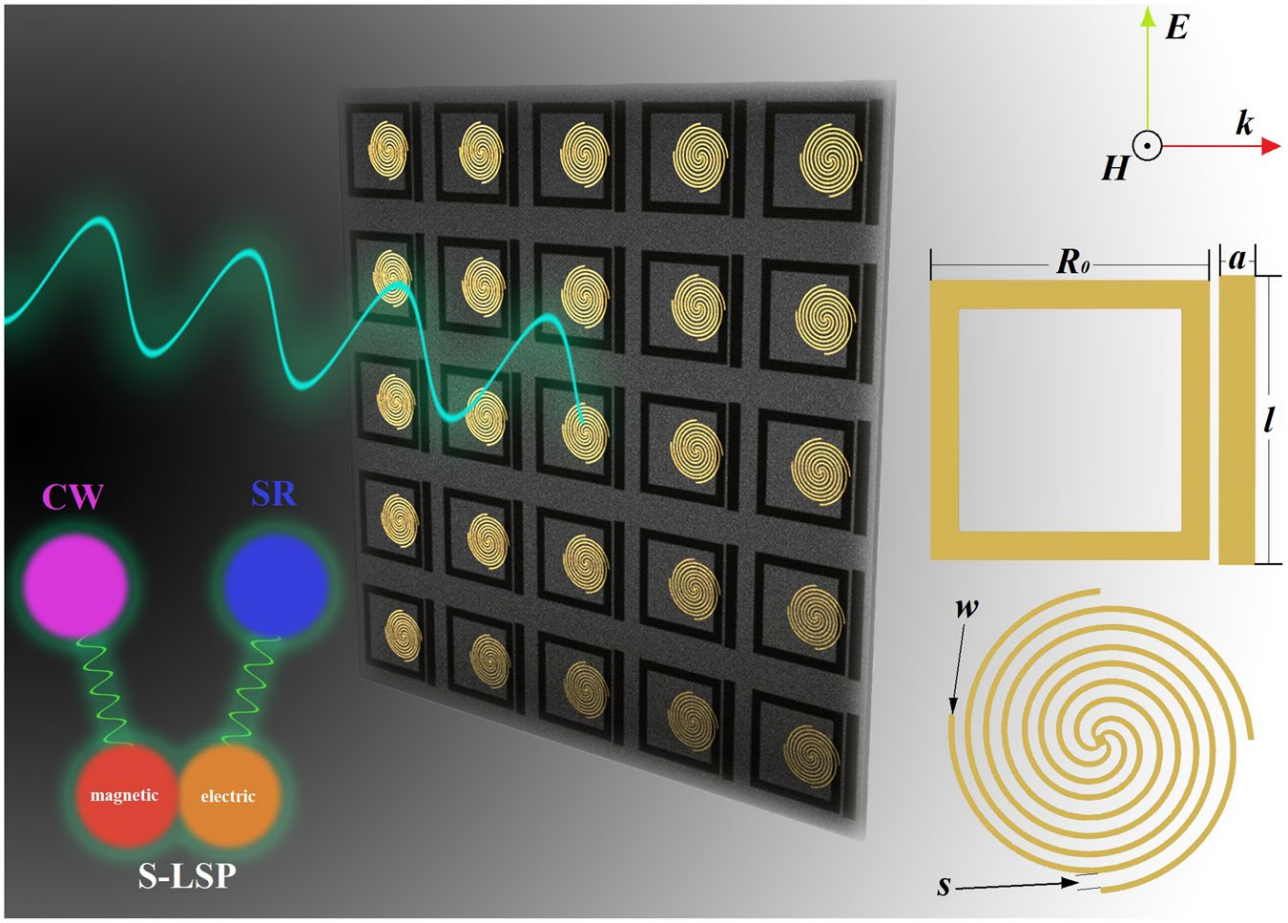
Schematics to the unit cell of bi-layered metamaterials for its front and back (created by the software of *Autodesk 3ds Max 20*1*6*, https://www.autodesk.com/products/3ds-max/overview).

The unit cell of our proposed bi-layered metamaterials structure is consisted of S-LSP (by the shape of clockwise 90° rotationally symmetric 4-arms spiral^24^) pure copper strips on the front of substrate and CW-SR hybrid pure copper strips on the back, as show in Fig. 1. The thickness of metallic structures is 0.035 *mm* which attaching on the surface of F4B dielectric slab with a thickness of ***t*** = 1.575 *mm* and a permittivity of 2.2. The full-periods of arranged periodic metamaterials sample is manufactured with 10×10 units.

The schematic of the unit cell of bi-layered metamaterials is illustrated in Fig. 1. We have adopted the geometrical parameters definitions of bi-layered metamaterial unit cell are given as follows: ***w*** = 0.2 *mm*, ***s*** = 0.4 *mm*, ***L*** = 24 *mm*, ***M*** = 10.8 *mm*, ***l*** = 18.2 *mm*, ***a*** = 2.3 *mm*, ***R*** = 14.1 *mm*, ***R***_***0***_ = 17.6 *mm*, what is occurred in simulation under the periodic boundary conditions. The substrate’s material is F4B, which is low dispersive and absorption while working below the terahertz frequencies. Numerical simulations are undertake using a finite element method. The simulation process of transmission curve is calculated in commercial software package *CST Microwave Studio*, with the plane electromagnetic wave incidence.

### Theoretical analysis and experiment results

**Figure 2:**
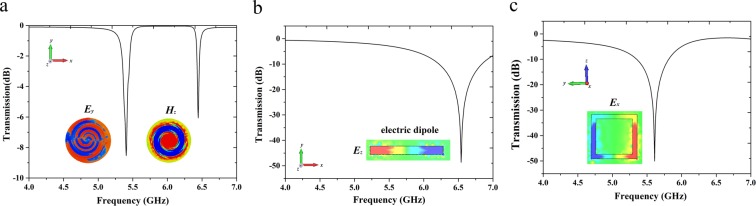
Transmission spectral of (**a**) electric S-LSP (*f*_E-SLSP_ = 5.405 GHz) and magnetic S-LSP (*f*_M-SLSP_ = 6.445 GHz) (without CW and SR), (**b**) CW (*f*_*CW*_ = 6.51 GHz) and (**c**) SR (*f*_*SR*_ = 5.585 GHz) under the condition of external fields excited directly (inset: field distributions for each dipoles).

To comprehensively explain the coupling mechanism of the proposed double-peaks mode A-EIT, we should primarily observe the transmission curves of excited S-LSP, CW and SR working alone. According to ref. ^24^, we set condition of external fields as the magnetic field vertical to the substrate plane and electric field parallel to the substrate plane, then removing the SR and CW from the substrate to get the transmission curve for S-LSP working alone be excited by external fields. In Fig. 2(a), there are two resonance dips at frequencies of 5.405 GHz and 6.445 GHz from the transmission curve for S-LSP alone. We know the S-LSP could be excited to produce simultaneously an electric dipole and a magnetic dipole by given condition of external fields (more pairs of electric dipole and magnetic dipole can be seen in enough wide frequency band) according to the electromagnetic feature of S-LSP. In order to distinguish which one is electric dipole and which one is magnetic dipole (frequency range of our investigation is limited to only one pair of electric and magnetic dipole be appeared), we investigate electric/magnetic field distributions at their corresponding frequencies of dipoles (5.405 GHz and 6.445 GHz), as shown in Fig. 2(a) insets. It can be observed that two pairs of equivalent electric currents flow along two curved routes on the metallic surface with opposite directions, resembling a electric dipole behavior. Furthermore, we can see the simulating ***E***_***y***_ field changes between negative and positive charges that making sure the electric dipole mode at 6.445 GHz, which is also similar to the molecular current that produces the electric dipole motions^33,35,36^. The ***H***_***z***_ distribution at 5.405 GHz presents the property of spirals and meanders. Similar to the above discussion for electric dipole, it is obvious this resonance at 5.405 GHz emerges by the magnetic S-LSP.

The necessary conditions for obtaining A-EIT are that the frequency difference between the two modes is small and the difference of their Q-value is large^37,38^. In order to achieve A-EIT phenomenon, we also need to utilize two extra coupling modes to acquire a pair of resonances that respective coupling to magnetic S-LSP (magnetic dipole) and electric S-LSP (electric dipole) from the excited S-LSP. Moreover, the different coupling ways to induce A-EIT (excite by external fields or near field coupling) unable to alter the type of dipoles and their resonance frequencies. We adopt a CW and SR structure adding on the back of the substrate meanwhile keeping the polarization direction of electric field to pointing the CW’s radial direction and polarization direction of magnetic field parallel to the substrate plane. By this direction of external field, the magnetic S-LSP is regards as dark mode which cannot be excited directly by external magnetic field but can be induced by electric resonance of CW. The CW’s electric resonance near field couples to the S-LSP will transform a magnetic resonance on the S-LSP. Therefore, After using CW and SR structure on the back of substrate, two electric dipoles from CW and SR are coupled separately to electric S-LSP and magnetic S-LSP as resonant modes. The frequencies of two electric dipole from CW and SR is *f*_*CW*_ = 6.51 GHz and *f*_*SR*_ = 5.585 GHz as shown in Fig. 2(b,c), and their Q-value are much lower than the electric dipole and magnetic dipole’s from S-LSP, respectively. According to the Fig. 2(b,c), two frequencies of electric dipoles from CW and SR are very close to the frequencies of S-LSP’s magnetic dipole and electric dipole, satisfy the necessary condition of generating A-EIT.

**Figure 3:**
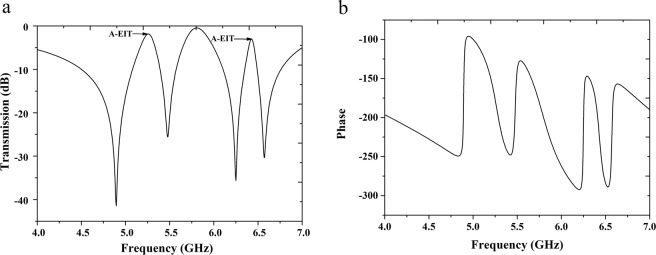
(**a**)The double-peaks mode A-EIT transmission numerical spectral at frequency of *f*_L_ = 5.255 GHz and *f*_R_ = 6.425 GHz (**b**) and its phase curve without wrap for whole unit cell of bi-layered metamaterials.

**Figure 4:**
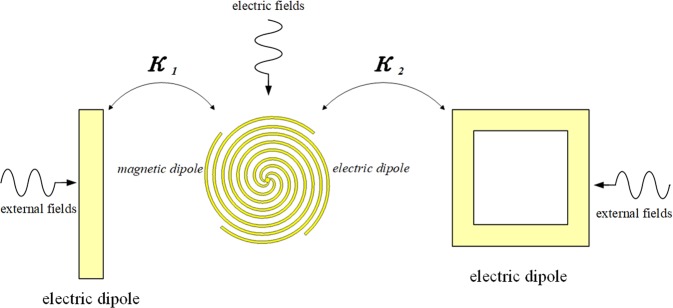
The schematic of energy coupling path for each modes (created by the software of *Visual Studio 2017*, https://visualstudio.microsoft.com/).

Now, we consider the transmission properties for the whole unit cell structure of bi-layered metamaterials which consisting of CW-SR hybrid and S-LSP. Under the effect of external fields according to upper right corner of Fig. 1, three electric resonances from the CW, SR and electric S-LSP are excited directly by the electric field. One bright-dark mode A-EIT occurs between in electric resonance caused by CW and magnetic S-LSP resonance from the CW near field couple to the S-LSP. Moreover, an A-EIT cannot occur between in two same type dipoles in general, such as in two electric resonances or two magnetic resonances. From the Fig. 2(a,c), the magnetic field on left and right side of SR are opposite but it is different interval for the electric S-LSP’s magnetic field, led to the amplitude of magnetic field of SR on left is larger than right’s, which is reason for the A-EIT occurring between in two same type dipoles for this structure. The differences of electric S-LSP and SR, magnetic S-LSP and CW’s resonance frequencies are △*f*_*1*_ = 0.18 GHz and △*f*_2_ = 0.065 GHz respectively which are far less than coherent frequencies of resonances themselves. The destructive interferences are between in bright-bright mode (SR and electric S-LSP) and bright-dark (CW and magnetic LSP) mode what having close frequencies will lead to two sharp A-EIT transmission peaks at frequencies of *f*_L_ = 5.255 GHz and *f*_R_ = 6.425 GHz, as shown in Fig. 3(a). So double-peaks mode A-EIT is successfully realized by introducing CW-SR hybrid and S-LSP bi-layered metamaterials structure and schematic of energy coupling path for each mode as shown in Fig. 4. Moreover, it can be clearly seen that an extra transmission peak at frequency of *f*_M_ = 5.805 GHz among the two A-EIT peaks at *f*_L_ and *f*_R_. To confirm whether this extra peak is an A-EIT transparency window, we have examined the phase curve without wrap of corresponding frequencies for the three transmission peaks, as shown in Fig. 3(b). At *f*_M_, the phase drastic change is too weak that unable to satisfy characteristic of A-EIT phenomenon. However, comparing to the other two peaks for *f*_L_ and *f*_R_’s exist strength phase drastic change because both frequencies of CW and SR approach corresponding frequencies of magnetic S-LSP and electric S-LSP adequately. On the other hand, the resonance frequencies of CW and SR are not close. We should unfortunately deduce this resonance at *f*_M_ producing among *f*_L_ and *f*_R_ not an A-EIT peak.

The inference effectives in the EIT phenomenon can be similar to the interactions of mass-spring system. Based on this, we can equivalent the metamaterials structure to the oscillator equations for analyzing them in detail. Referring to oscillator-coupled system from the instructive works of predecessors^23,26,41^, the oscillator equations for coupling modes of proposed A-EIT what are based on the coupled Lorentz oscillator model being shown as follows:

1 $$\frac{{\partial }^{2}{S}_{{\rm{SR}}}(t)}{\partial {t}^{2}}+{\gamma }_{{\rm{SR}}}\frac{\partial {S}_{{\rm{SR}}}(t)}{\partial t}+{\omega }_{{\rm{SR}}}^{2}{S}_{{\rm{SR}}}(t)=-{\kappa }_{2}^{2}{S}_{{\rm{E}}-{\rm{SLSP}}}(t)-\frac{{q}_{SR}}{{m}_{SR}}{E}_{in}(t)$$

2 $$\frac{{\partial }^{2}{S}_{{\rm{CW}}}(t)}{\partial {t}^{2}}+{\gamma }_{{\rm{CW}}}\frac{\partial {S}_{{\rm{CW}}}(t)}{\partial t}+{\omega }_{{\rm{CW}}}^{2}{S}_{{\rm{CW}}}(t)=\frac{{q}_{{\rm{CW}}}}{{m}_{{\rm{CW}}}}{E}_{{\rm{in}}}(t)-{\kappa }_{2}^{2}{S}_{{\rm{M}}-{\rm{SLSP}}}(t)$$

3 $$\frac{{\partial }^{2}{S}_{{\rm{M}}-{\rm{SLSP}}}(t)}{\partial {t}^{2}}+{\gamma }_{{\rm{M}}-{\rm{SLSP}}}\frac{\partial {S}_{{\rm{M}}-{\rm{SLSP}}}(t)}{\partial t}+{\omega }_{{\rm{M}}-{\rm{SLSP}}}^{2}{S}_{{\rm{M}}-{\rm{SLSP}}}(t)=-{\kappa }_{1}^{2}{S}_{{\rm{CW}}}(t)$$

4 $$\frac{{\partial }^{2}{S}_{{\rm{E}}-{\rm{SLSP}}}(t)}{\partial {t}^{2}}+{\gamma }_{{\rm{E}}-{\rm{SLSP}}}\frac{\partial {S}_{{\rm{E}}-{\rm{SLSP}}}(t)}{\partial t}+{\omega }_{{\rm{E}}-{\rm{SLSP}}}^{2}{S}_{{\rm{E}}-{\rm{SLSP}}}(t)=\frac{{q}_{{\rm{E}}-{\rm{SLSP}}}}{{m}_{{\rm{E}}-{\rm{SLSP}}}}{E}_{{\rm{in}}}(t)-{\kappa }_{2}^{2}{S}_{{\rm{SR}}}(t)$$

5 $$\begin{array}{c}{\chi }_{eff}=\frac{{q}_{SR}{S}_{SR}+{q}_{CW}{S}_{CW}+{q}_{E-LSP}{S}_{E-LSP}+{q}_{M-LSP}{S}_{M-LSP}}{{\varepsilon }_{0}{E}_{in}}\\ =C\frac{{\omega }^{2}-{\omega }_{0}^{2}+j\omega {\gamma }_{M-SLSP}}{{({\kappa }_{1}{\kappa }_{2})}^{4}-({\omega }^{2}-{\omega }_{0}^{2}+j\omega {\gamma }_{CW})({\omega }^{2}-{\omega }_{0}^{2}+j\omega {\gamma }_{SR})({\omega }^{2}-{\omega }_{0}^{2}+j\omega {\gamma }_{M-SLSP})({\omega }^{2}-{\omega }_{0}^{2}+j\omega {\gamma }_{E-SLSP})}\end{array}$$

6 $$T=|\frac{4\sqrt{1+{\chi }_{eff}}}{{(1+\sqrt{1+{\chi }_{eff}})}^{2}{e}^{j(2\pi d/\lambda )\sqrt{1+{\chi }_{eff}}}-{(\sqrt{1+{\chi }_{eff}}-1)}^{2}{e}^{-j(2\pi d/\lambda )\sqrt{1+{\chi }_{eff}}}}|$$

In above equations, *S*_*i*_, *q*_*i*_, *m*_*i*_, *γ*_*i*_*, к*_*i*_ (*i* is CW, SR, electric S-LSP or magnetic S-LSP) are the effective displacement, effective charge, effective mass, loss factor and coupling strength. *χ*_*eff*_ is linearly polarization and *T* is transmission coefficient. They are only dictated by the shape, geometric parameters and materials of structure that independent of the external fields. The incident wave for frequency domain can be expressed by ${E}_{in}(t)={E}_{in}(\omega ){e}^{-j\omega t}$ and ${H}_{in}(t)={H}_{in}(\omega ){e}^{-j\omega t}$.

In Eq. (1), both SR and electric S-LSP can be excited by the electric field, so equation right exists the field component. For the same reason in Eq. (2), CW can be excited directly by the electric field that equation right including the electric field component. In Eq. (3), the magnetic S-LSP cannot be excited by external field that only be near field coupled by the CW, so the equation right only including the coupling component from the CW. Finally, the electric S-LSP and SR can also be excited directly by electric field and partial energy exchanges between electric-SLSP and SR, it is reason for the Eq. (4) right inducing the electric field and SR components. According to the theory of higher order differential equations, we have solved the expression of *χ*_*eff*_ and *T* by Eqs. (5) and (6).

**Figure 5:**
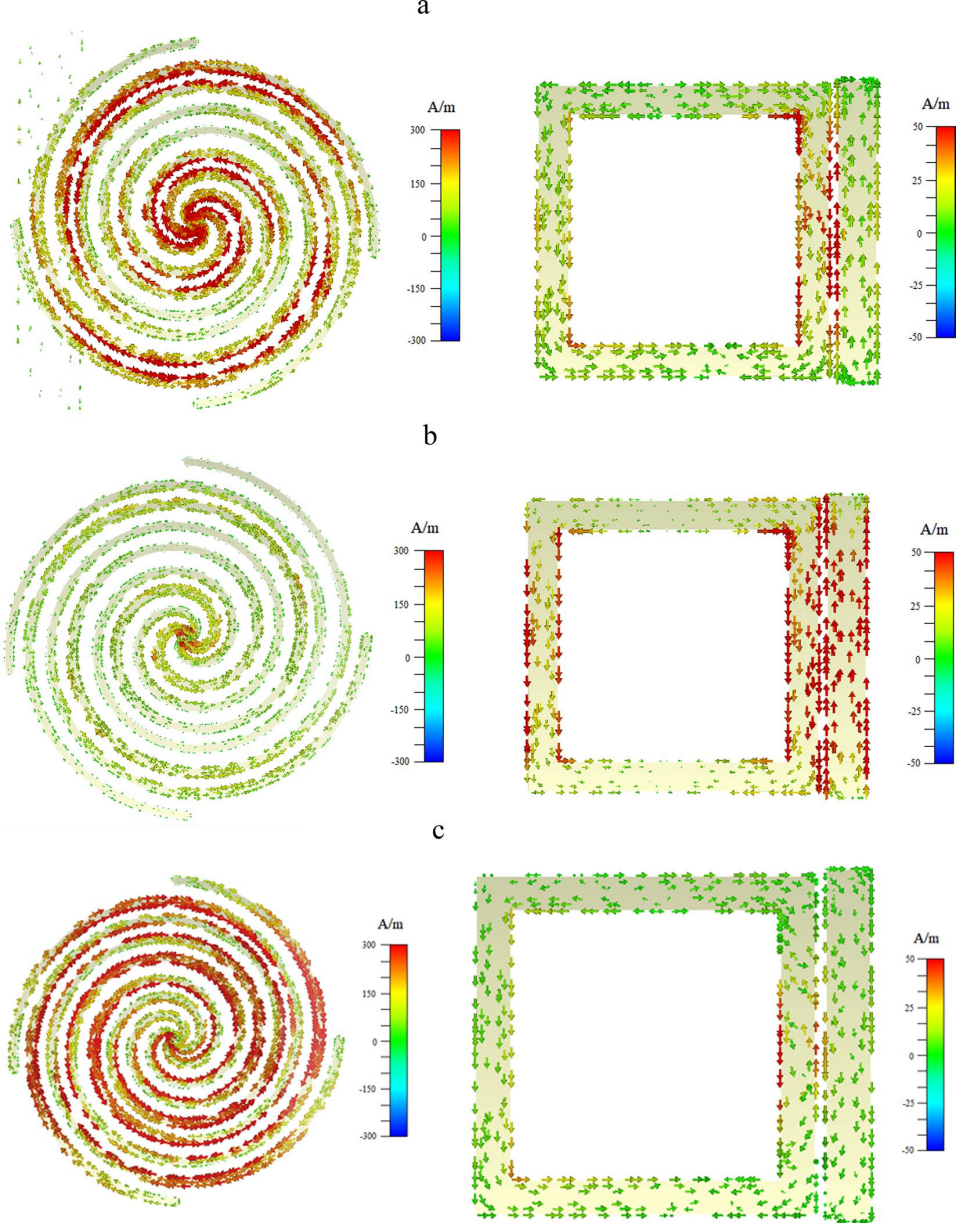
The surface current distributions for three transmission peaks at their frequencies of (**a**) *f*_*L*_, (**b**) *f*_*M*_ and (**c**) *f*_*R*_ (created by the software of *CST Microwave Studio 2018*, http://www.cst.com/).

To further understand the behavior of double-peaks mode A-EIT, we have investigated the surface current distributions for the frequencies of three transmission peaks of A-EIT as shown in Fig. 5(a–c) respectively. At the three frequencies of transmission peaks, it can be found that most of the electromagnetic energy concentrating on S-LSP that indicates the S-LSP near field coupling from the CW and SR, then the CW and SR do not play a very important role. It indicates that the destructive interference existing between in electric and magnetic dipole that can be explained by the anti-bonding modes^40,42,43^, indicating the reason of the disappearing dipole moment excited from the SR. The orders to the amplitude for surface current density on each transmission peaks are *f*_R_ > *f*_*SR*_ > *f*_*M*_, which consistent with the orders of their Q-value and extent of phase changes. The distributions of surface currents for S-LSP, CW and SR also like electric dipoles and magnetic dipoles’ moments, and the direction of magnetic dipoles from the S-LSP are opposite for the SR’s. So that surface current distributions sufficiently prove the transmission curve is from the double-peaks mode A-EIT exciting.

**Figure 6:**
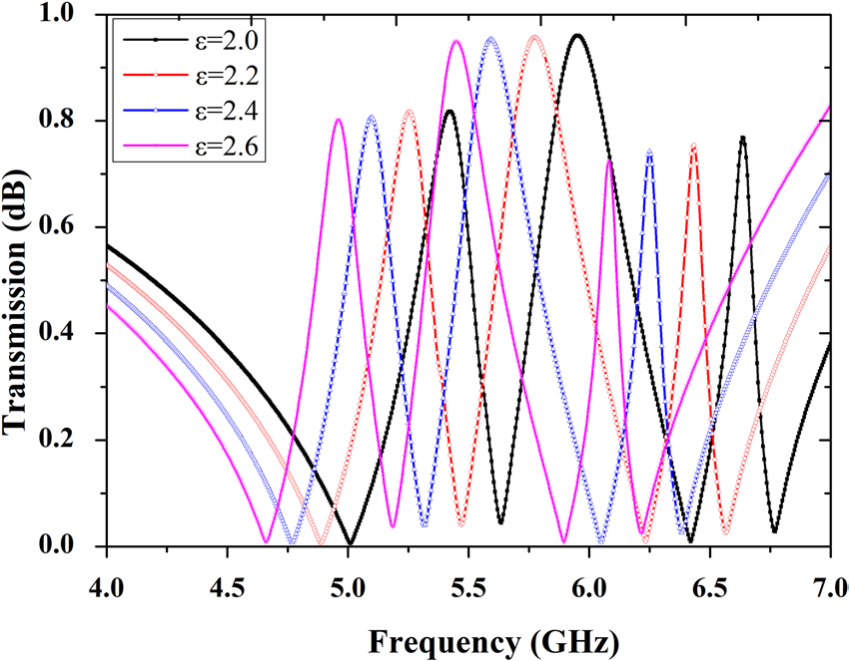
Numerical transmission curve shifts of the A-EIT metasurface structure respect to changes for permittivity of the substrate.

We numerically calculate the effect of the transparency window for the different permittivity. It can be observed a clear red-shift of the transmission curve when increasing the permittivity of the substrate from 2.0 to 2.6 as shown in Fig. 6. Owing to the feature of A-EIT transmission spectra changes linearly with the dielectric, this structure also can be regarded as a permittivity sensor at free space. We can foreseeable that the frequencies of transmission peaks depend on the dielectric properties that has potential application on permittivity sensor.

**Figure 7:**
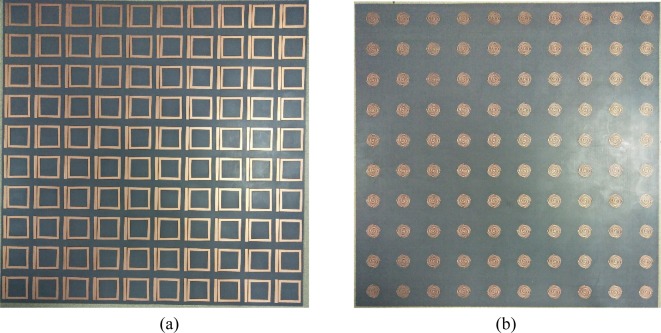
The front (**a**) and back (**b**) side of bi-layered metamaterials manufactured sample for the experiment.

**Figure 8:**
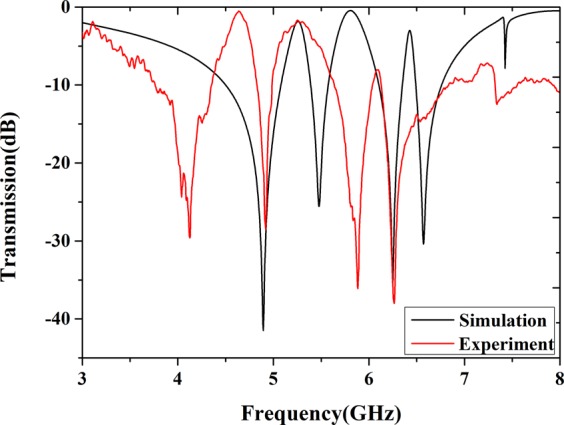
The experimental result (red curve) and simulation (black curve) of transmission curve for the sample.

At last, we have manufactured the bi-layered metamaterials sample for the experiment. The front side is consist of 10 × 10 CW-SR hybrids, then the back is consist of 10×10 S-LSPs as shown in Fig. 7(a,b). The sample is measured by a pair of X-band horn antennas and vector network analyzer in anechoic chamber. From the Fig. 8, it can be clearly observed three transmission peaks from the experimental results which is good agreement with the simulation transmission curve, but slight drifts in Q-value of three transmission peaks and some frequencies deviation existing. We assume the reason for the errors is due to the uncertainty of dielectric and limited manufacture level of the sample. So experimental evidence of the existence for double-peak A-EIT in the microwave regime have been described.

## Conclusion

In conclusion, we had designed a A-EIT with double transparent peaks in the spectrum based on plane double-layered metasurface structure composing of S-LSP and CW-SR hybrid. The proposed structure simultaneously utilizes electric and magnetic dipole originating from the S-LSP which be used as the coupling modes to achieve double-peaks mode A-EIT. Furthermore, we analyzed the surface current distributions at the frequencies of three transmission peaks and their equivalent Lorentz model had been established. The experiment results matched well to the simulations. The double-peaks mode A-EIT provides a kind of ways to design the optical computing and communications in highly integrated optical circuits frequency selective, slow light and width band fitters in the microwave regime.

## References

[1] Imamoğlu A, Boller KJ, Harris SE (1991). Observation of electromagnetically induced transparency. Physical Review Letters.

[2] Harris SE (1997). Electromagnetically induced transparency. Physics Today.

[3] U R. Gabinet. & C. O. Osuji. Optical materials and metamaterials from nanostructured soft matter, 12(9): 2172–2183, Nano Research (2019).

[4] Vasilantonakis, N. *et al*. “Refractive index sensing with hyperbolic metamaterials: strategies for biosensing and nonlinearity enhancement.” Optics Express 23.11,14329 (2015). 10.1364/OE.23.014329 26072797

[5] Cong L (2015). Experimental demonstration of ultrasensitive sensing with terahertz metamaterial absorbers: a comparison with the metasurfaces. Applied Physics Letters.

[6] Sakai O, Maeda J, Shimomura T, Urabe K (2013). Functional composites of plasmas and metamaterials: flexible waveguides, and variable attenuators with controllable phase shifta. Physics of Plasmas.

[7] Mohammad. M, Eunjong. K, Ferreira. VS (2018). Superconducting metamaterials for waveguide quantum electrodynamics. Nature Communications.

[8] Qingxia M, Hao L, Xia H (2018). & Xin yu, Z. Microscopic–macroscopic entanglement transfer in optomechanical system: non-markovian effects. Optics Communications.

[9] Bagci F, Akaoglu B (2017). Single and multi-band electromagnetic induced transparency-like metamaterials with coupled split ring resonators. Journal of Applied Physics.

[10] Liang DC (2017). Plasmonic analogue of electromagneticlly induced transparency in stereo metamaterials. IEEE Journal of Selected Topics in Quantum Electronics.

[11] Nakanishi, T., & Kitano, M., Storage and retrieval of electromagnetic waves using electromagnetically induced transparency in a nonlinear metamaterial. Applied Physics Letters, 112(20) (2018).

[12] Jenkins SD, Ruostekoski J (2013). Metamaterial transparency induced by cooperative electromagnetic interactions. Physical Review Letters.

[13] Cao W, Singh R, Zhang C, Han J (2013). Plasmon-induced transparency in metamaterials: active near field coupling between bright superconducting and dark metallic mode resonators. Applied Physics Letters.

[14] Tian Ma (2019). All-dielectric metamaterial analogue of electromagnetically induced transparency and its sensing application in terahertz range. Opt. Express..

[15] Han S (2016). Tunable electromagnetically induced transparency in coupled three-dimensional split-ring-resonator metamaterials. Sci. Rep.

[16] Wu C, Khanikaev AB, Shvets G (2011). Broadband Slow Light Metamaterial Based on a Double-Continuum Fano Resonance. Phys. Rev. Lett..

[17] Arju N (2015). Optical Realization of Double-Continuum Fano Interference and Coherent Control in Plasmonic Metasurfaces. Phys. Rev. Lett..

[18] Taubert R, Hentschel M, Giessen H (2013). Plasmonic analog of electromagnetically induced absorption: simulations, experiments, and coupled oscillator analysis. Journal of the Optical Society of America B Optical Physics.

[19] Thuy, V. T. T. *et al*. Quasi-dark mode in a metamaterial for analogous electromagnetically-induced transparency. 12(11), 115102:1-9, Physics (2010).

[20] Monika DK (2017). Plasmon induced transparency effect through alternately coupled resonators in terahertz metamaterial. Opt. Express.

[21] Gwo S, Lin MH, He CL, Chen HY, Teranishi T (2012). Bottom-up assembly of colloidal gold and silver nanostructures for designable plasmonic structures and metamaterials. Langmuir.

[22] Dao, T. D. *et al*. An on‐chip quad‐wavelength pyroelectric sensor for spectroscopic infrared sensing. vol 6, 1900579, 1-9, Advanced Science (2019). 10.1002/advs.201900579 PMC6794626 31637158

[23] Dietze D (2013). Ultrastrong coupling of intersubband plasmons and terahertz metamaterials. Appl. Phys. Lett.,.

[24] Huidobro PA (2014). Magnetic localized surface plasmons. Phys.rev.x.

[25] Zhao Z, Chen Y, Gu Z, Shi W (2018). Maximization of terahertz slow light by tuning the spoof localized surface plasmon induced transparency. Optical Materials Express.

[26] Ooi K, Okada T, Tanaka K (2011). Mimicking electromagnetically induced transparency by spoof surface plasmons. Physical Review B Condensed Matter.

[27] He Y, Hao Z, Yi J, He S (2011). Plasmon induced transparency in a dielectric waveguide. Applied Physics Letters.

[28] Aristi Christofi (2018). Giant enhancement of Faraday rotation due to electromagnetically induced transparency in all-dielectric magneto-optical metasurfaces. Opt. Lett..

[29] Taubert, R. *et al*. Classical Analog of Electromagnetically Induced Absorption in Plasmonics. *Nano Lett.* **12**(3), 1367–1371 (2013). 10.1021/nl2039748 22273467

[30] Taubert R, Hentschel M, Giessen H (2013). Plasmonic analog of electromagnetically induced absorption: simulations, experiments, and coupled oscillator analysis. Journal of the Optical Society of America B Optical Physics.

[31] Cetin AE, Artar A, Turkmen M, Yanik AA, Altug H (2011). Plasmon induced transparency in cascaded π-shaped metamaterials. Optics Express.

[32] Xu, Z., Liu, S., Li, S. & Yin, X. Analog of electromagnetically induced transparency based on magnetic plasmonic artificial molecules with symmetric and antisymmetric states. *Phys. Rev. B* **041104**, 99(4), (2019).

[33] Matsui T (2017). A brief review on metamaterial-based vacuum electronics for terahertz and microwave science and technology. Journal of Infrared Millimeter & Terahertz Waves.

[34] Pors A, Moreno E, Martin-Moreno L, Pendry JB, Garcia-Vidal FJ (2012). Localized spoof plasmons arise while texturing closed surfaces. Physical Review Letters.

[35] Liao Z, Luo GQ, Cai BG, Pan BC, Cao WH (2019). Subwavelength negative-index waveguiding enabled by coupled spoof magnetic localized surface plasmons. Photonics Research.

[36] Podgornov FV, Haase W (2018). Chiroptic response of ferroelectric liquid crystals triggered with localized surface plasmon resonance of achiral gold nanorods. Applied Physics Letters.

[37] Thanvanthri S, Wen J, Rubin MH (2005). Effects of mismatched transmissions on two-mode squeezing and EPR correlations with a slow light medium. Physical Review A.

[38] Yu X, Zhang J (2010). Multi-normal mode-splitting for an optical cavity with electromagnetically induced transparency medium. Optics Express.

[39] Khanikaev AB (2012). Electromagnetically induced polarization conversion. Opt. Comm..

[40] Alonso-González P (2013). Visualizing the near-field coupling and interference of bonding and anti-bonding modes in infrared dimer nanoantennas. Optics Express.

[41] Sun C, Li H, Gong Q, Chen J (2016). Ultra-small and broadband polarization splitters based on double-slit interference. Applied Physics Letters.

[42] Yang X (2014). Tunable ultracompact chip-integrated multichannel filter based on plasmon-induced transparencies. Applied Physics Letters.

[43] Saadat S, Adnan M, Mosallaei H, Afshari E (2013). Composite metamaterial and metasurface integrated with non-foster active circuit elements: a bandwidth-enhancement investigation. IEEE Transactions on Antennas and Propagation.

